# Contribution of hospitals and clinical services to global warming: a scoping systematic review

**DOI:** 10.3389/fpubh.2026.1778269

**Published:** 2026-05-12

**Authors:** Waldo Merino-Urrutia, Claudio Cárcamo-Fuentes, Mauricio Peña, Badra Zalej-Rakela, Joaquín Rodríguez-García, María de los Ángeles Gottschalk-Cuevas, Francisco Rubilar-Rocha, María José Martínez-Zapata

**Affiliations:** 1 Facultad de Medicina, CIGES, Universidad de La Frontera, Chile; 2 Nursing Department, Universidad de La Frontera, Chile; 3 Hernán Henríquez Aravena Hospital, Temuco, Chile; 4 Facultad de Medicina, Universidad de La Frontera, Chile; 5 Facultad de Medicina, Universidad Mayor Santiago, Chile; 6 Instituto de Medio Ambiente, Universidad de La Frontera, Chile; 7 Iberoamerican Cochrane Center, IR Sant Pau, Barcelona. CIBERESP, Madrid, Spain

**Keywords:** anesthesia, carbon footprint, climate change, global warming, greenhouse gas, health care, hospitals, waste products

## Abstract

**Introduction:**

This Scoping Review aims to conduct an up-to-date and comprehensive search of the scope of existing studies on how hospitals and clinical services contribute to global warming.

**Methods:**

Data sources: PubMed, Scopus and Embase. Selection criteria: Studies published from January 2000 to December 2024, in all languages and of any design type. Exclusion criteria: Secondary studies. Guidelines or recommendations. Letters or comments without new data. Studies of hospital products carried out in non-hospital environments. Outpatient units. Publications comparing hospital activities with extra-hospital services that differ only by transport. Primary outcome measure. The contribution to climate change of hospitals and clinical services, through their processes and activities. Data extraction and analysis. Three authors independently selected the articles according to the study objectives. If there were differences, these were resolved through discussion. The same method was applied for data extraction.

**Results:**

The literature search yielded 905 results, excluding duplicates. 184 studies were included in the scoping review. The studies were grouped into the following areas: anesthetic technique, medical devices, surgical procedures, other clinical activities, waste management, support units, and hospitals. A description is also made of the hospital processes involved in the generation of greenhouse gas emissions, such as incineration, laundry, among others. The most numerous publications were related to anesthesiology, devices and operating room. 13.6% of the studies are either experimental or quasi-experimental. Thirteen studies incorporated economic aspects, mainly description of costs. We did not find any studies that carried out a sustainability analysis, in terms of the relationship between costs, emissions and clinical effectiveness.

**Conclusion:**

In conclusion, this study provided a comprehensive overview of hospitals’ contribution to greenhouse gas emissions and other environmental impacts. We did not find any studies that carried out a sustainability analysis, in terms of the relationship between costs, emissions and clinical effectiveness. Research that incorporates economic aspects and sustainability studies is necessary for the implementation of effective actions.

## 1 Introduction

Human activities at both individual and societal levels contribute substantially to environmental degradation, which has measurable effects on human health. Exposure to climate change-related environmental stressors has been linked to an increased incidence of infectious diseases, respiratory morbidity, and all-cause mortality (1). The healthcare sector was recognized as a major contributor of contaminating emissions, accounting for 5.2% of global greenhouse gas (GHG) emissions in 2019 (2). In 2018, the health industry in the United States alone produced 1,692 kg CO_2_e (carbon dioxide equivalent) per capita and contributed to a loss of between 123,000 and 388,000 years of life adjusted for disability (3, 4). Among these, hospitals are the largest contributors, responsible for 39% of the sector’s emissions (5).

The Declaration of Helsinki emphasizes the importance of safeguarding human and planetary health and highlights the urgent need for sustainable strategies in the healthcare sector (6, 7). This raises an important question: What evidence exists regarding the role of healthcare services and hospitals in contributing to global warming? Additionally, what factors play a significant role in this phenomenon? Which interventions are supported by evidence?

The exploration of the impact of clinical services and hospitals on GHG emissions and their contribution to global warming yields a plethora of publications with varying objectives, approaches, resources, and measurement techniques. A comprehensive overview of the existing literature is necessary, and a scoping review is particularly useful when the available evidence is heterogeneous and cannot be precisely synthesized (8–11). This type of study effectively describes the range and nature of the available evidence on a specific topic, identifying both areas that have been studied and those that require further research. This scoping review seeks to provide an updated and thorough examination of the existing studies on the contributions of hospitals and clinical services to global warming.

## 2 Materials and methods

This scoping review was carried out following the recommendations for reporting systematic reviews and meta-analyses extension for scoping reviews (PRISMA-ScR) (12) and for the extraction, analysis, and presentation of results in scoping reviews (8, 9, 13). The study protocol has previously been published (14).

The objective here was to map and synthesize the existing evidence on the contribution of hospitals and clinical services to climate change through their processes, activities, and diagnostic and therapeutic practices, guided by the Population-Concept-Context (PCC) framework: hospitals (population), greenhouse gas emissions (concept), and hospital activities and processes (context). The search strategy was comprehensive and extensive, aiming to identify well-researched, under-researched, and underdeveloped areas related to the impact of hospitals and clinical services on global warming.

Published primary studies of any design or language that assessed the impact of hospital activity on global warming were included. Letters were included if they presented original data from the respective clinical centers. To ensure the reliability of the carbon emission measurements, only studies published in peer-reviewed journals were considered.

In contrast to the protocol, secondary studies, including systematic and narrative reviews, were excluded to prioritize primary sources of evidence and enhance their relevance. Also excluded were guidelines, standards, and recommendations; letters or commentaries lacking original data; studies focused on waste management rather than carbon footprint or life cycle assessment; research conducted in laboratory settings or studies that estimated or simulated product use without being performed in hospital environments or under hospital conditions; studies involving outpatient units operating outside hospital settings; hospital interventions that assessed only patient transportation (i.e., travel-related emissions); studies on emissions from health services or governmental organizations that did not clearly specify the contribution attributable to hospitals; and comparisons between hospital-based activities and similar non-hospital services when transport was the sole differentiating factor.

The review focused on studies published between January 2000 and December 2024, utilizing the PubMed, Scopus, and Embase literature databases. In general, the search terms included “carbon footprint,” “greenhouse effect,” “greenhouse gas,” “global warming,” “climate change,” and “life cycle assessment.” These terms were truncated with keywords such as “hospitals,” “laboratories,” “gases,” “anesthesia,” “analgesia,” “waste products,” and “health care.” The specific search strategies for each database are detailed in Supplementary material 1.

The research team agreed on the working method to ensure standardization and precision in study screening and data extraction. According to the protocol, three authors independently selected the articles by title and abstract. First, an initial search was conducted using keywords from the title and abstract. Subsequently, the authors checked the texts to ensure the studies met the selection criteria, and additional studies were sought from the reference lists of the selected studies. An article was automatically included in those cases where two or three authors agreed with the selected published study. Any differences were resolved by consensus through peer discussion. In case of disagreement between peers, the third author resolved the conflict. The same method was applied for data extraction. Regular team meetings were held throughout the data extraction process to discuss progress and refine the data extraction.

The extracted outcomes included title, author, publication date, journal, study type/design, objectives, methodology, health services/hospital characteristics, data source, way of estimating GHG emissions, study population, context, and key findings. When reported, carbon footprint (CF) and emission sources, such as water consumption, heating, and cooling, were also recorded.

## 3 Results

**Figure 1:**
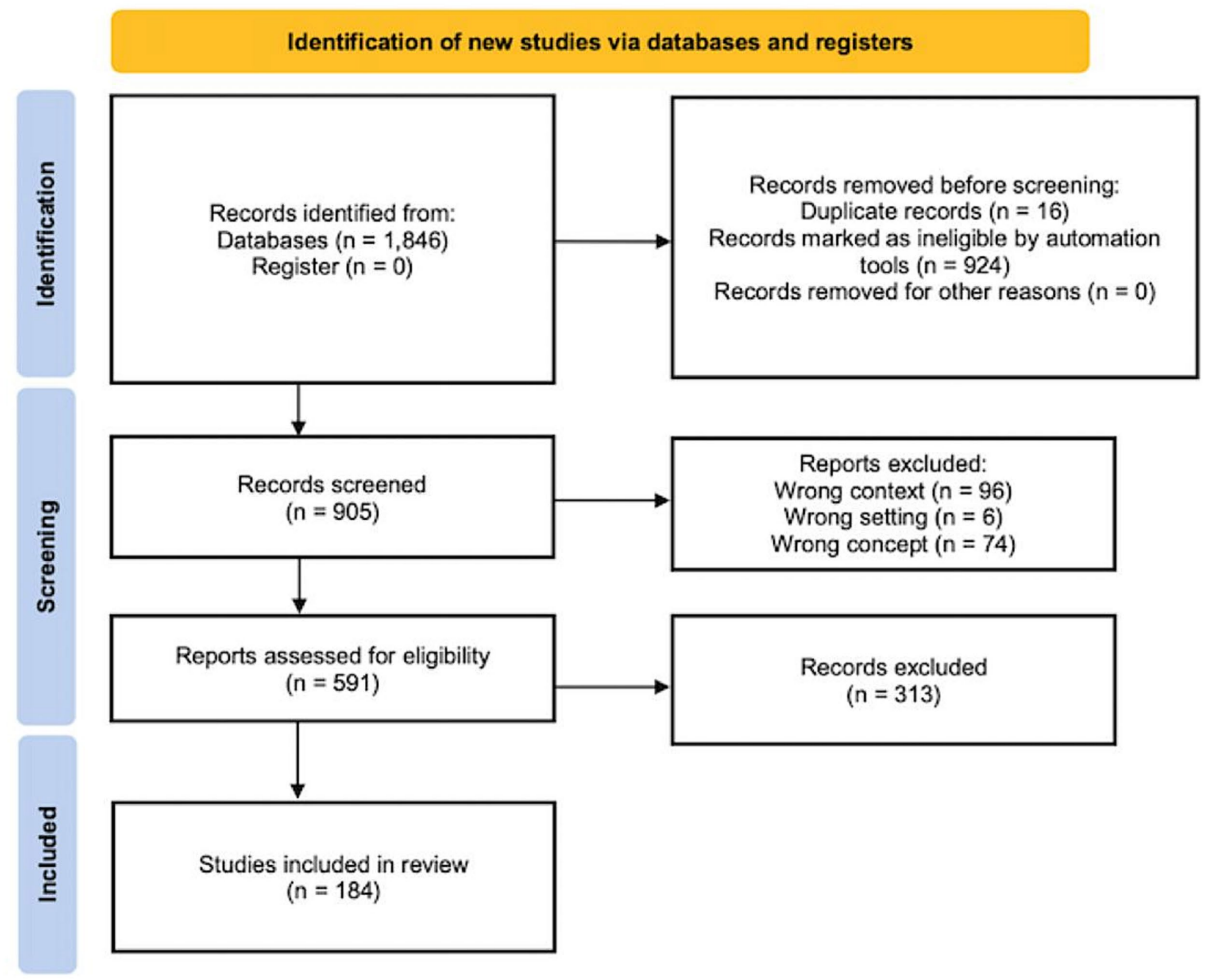
PRISMA flow diagram.

The bibliographic search yielded 905 results, excluding duplicates. After excluding 314 studies based on their titles and abstracts, 591 publications were assessed in full text, of which 407 did not meet the eligibility criteria. Finally, 184 studies were included in the review (Figure 1) (15).

**Figure 2:**
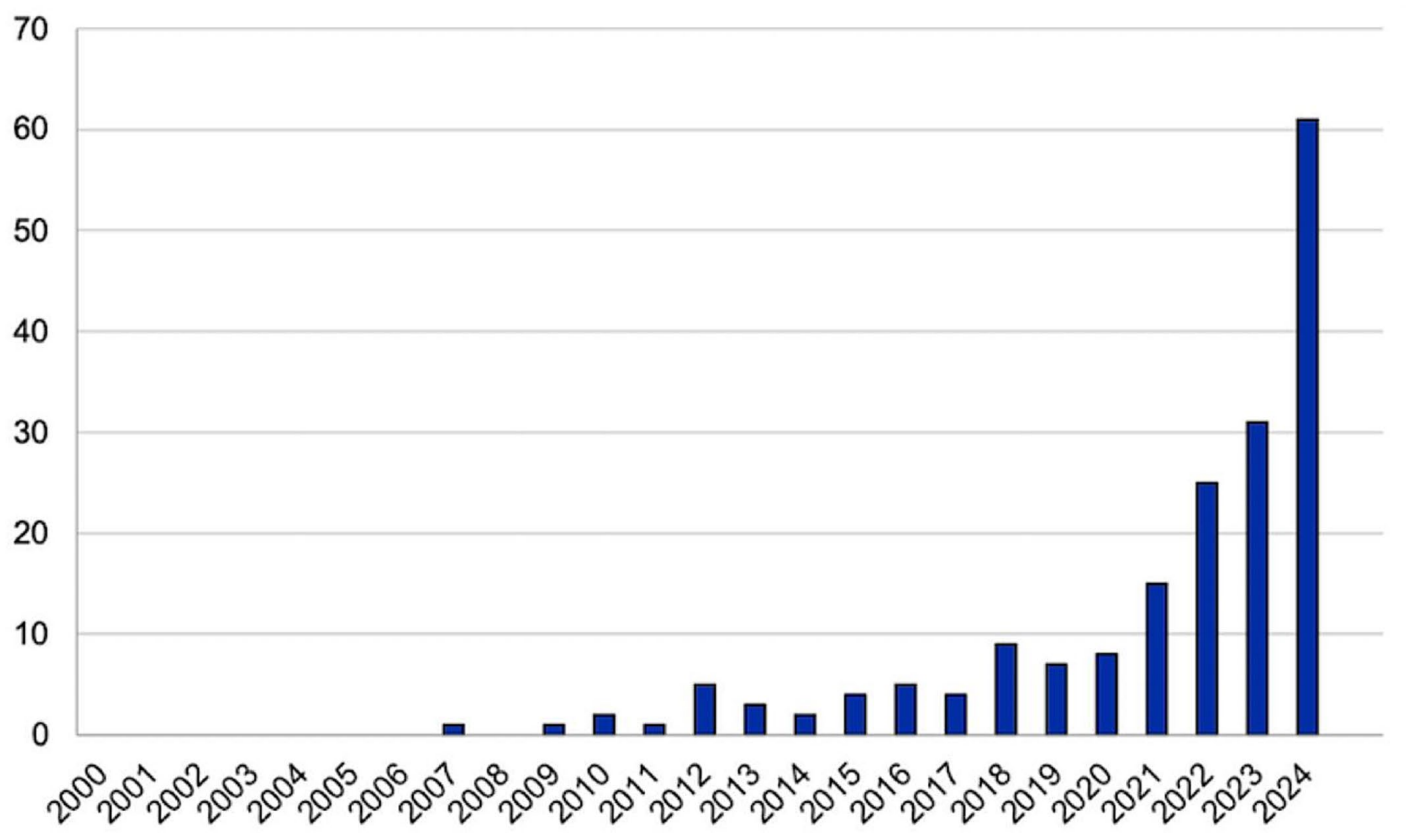
Number of publications per year.

**Figure 3:**
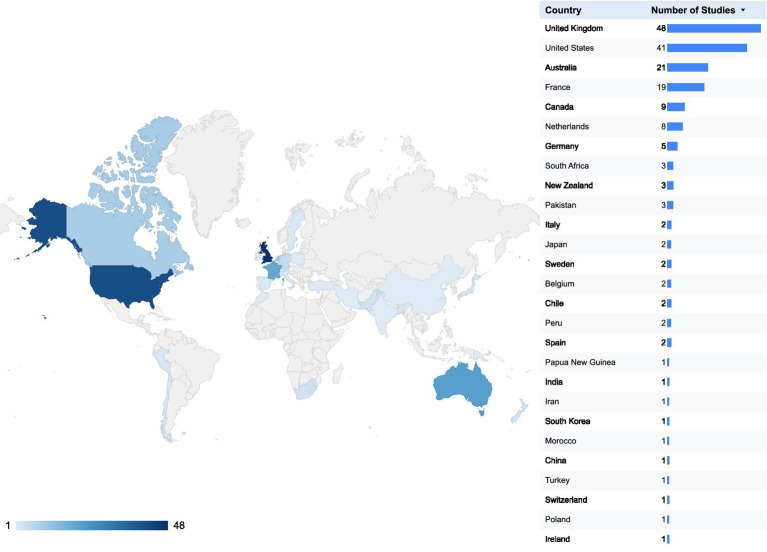
Distribution of the number of publications by country.

Since 2000, there has been a progressive increase in the number of published studies, particularly noted in 2012, 2018, and 2024 (Figure 2). Publications are predominantly from English-speaking countries, with the UK leading with 48 studies (Figure 3) (16).

An important aspect to highlight is the estimation of the CF. The most common method for calculating CO_2_ equivalent emissions from resource consumption is the use of conversion factors, which were applied in 74 studies (40.2%). Additionally, 58 (31.5%) studies conducted a life cycle analysis, 18 (9.8%) studies conducted a GHG protocol, and 14 (7.6%) studies used other methodologies. However, 20 (10.9%) studies reported GHG emissions figures without specifying how these estimates were calculated.

Of the published studies, 134 (72.8%) were original research articles, 33 (17.9%) were posters, and 17 (9.4%) were letters to the editor. A high prevalence of retrospective studies was noted. Most studies were descriptive, and only 13.6% were experimental or quasi-experimental studies; most were before/after comparisons.

**Figure 4:**
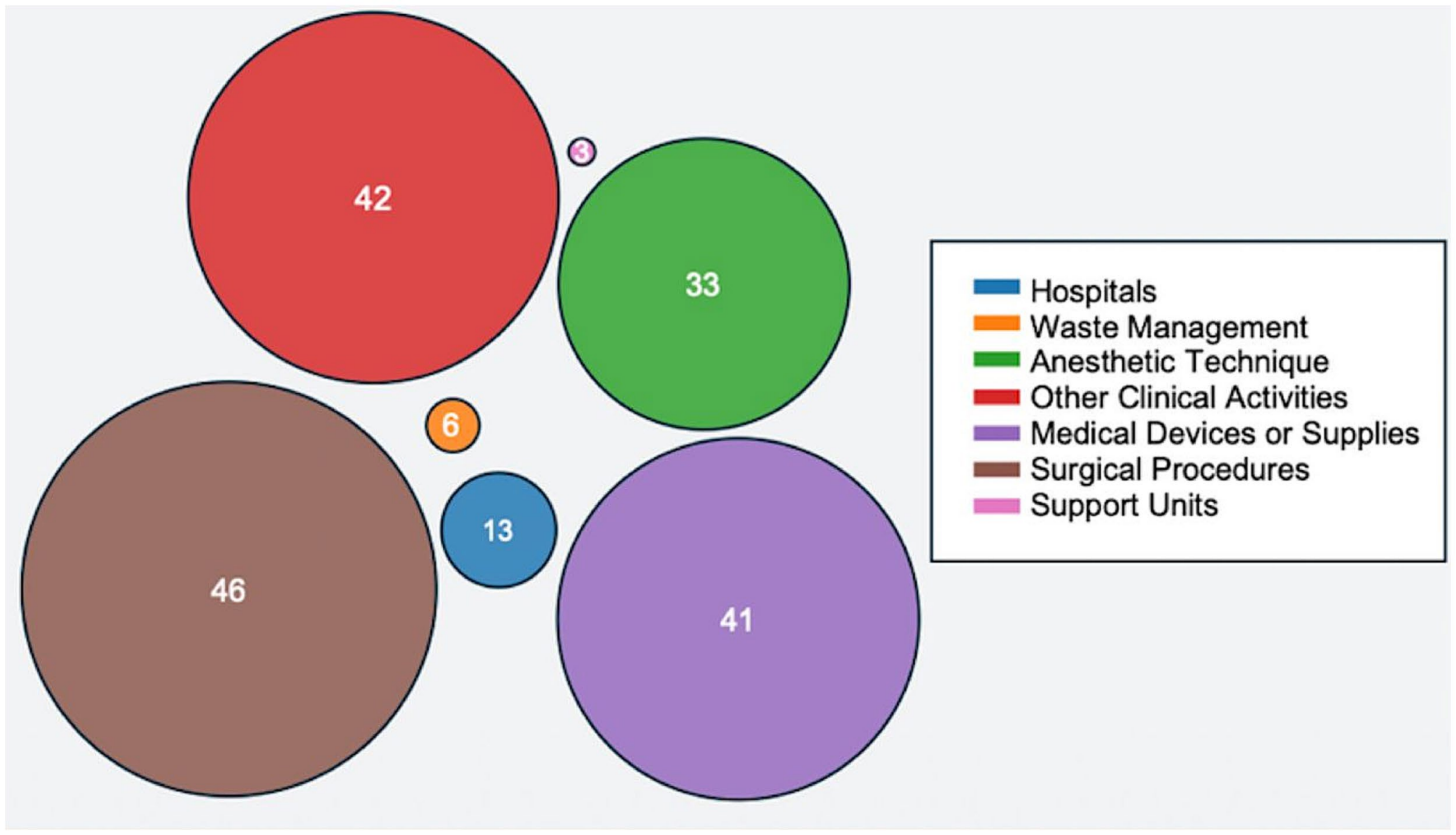
Volume of studies published from sectors that generate emissions.

Seven major emission-generating sectors were identified as follows (Figure 4).

### 3.1 Anesthetic technique

In this area, most *studies focused on describing and quantifying the use of anesthetic agents* and their environmental impact in clinical practice settings. Based on these studies, anesthetic gases, particularly desflurane and nitrous oxide (N_2_O), are the most significant contributors to emissions compared to alternative anesthetic techniques such as total intravenous anesthesia (TIVA) and regional anesthesia (17–26) (Table 1).

**Table 1:** Studies focused on describing and quantifying the use of anesthetic agents and their emissions.

Ref	Authors	Year	Emissions	Centers
(17)	MacKenzie, L et al	2018	25 general anesthetics performed, the emissions was 1600.8 KgCO₂e, approximately 200 tonnes per year.	Princess Royal University Hospital, King’s College Hospital NHS Trust
(18)	Kennedy, R et al	2020	30,000 operations annually. The GWP of vapor used has fallen from 373 tonnes of CO₂ e in 2012/13 to 102 tonnes for 2018/19. Most of this reduction is decreasing use of desflurane.	Christchurch Hospital, New Zealand
(19)	Weinberg, L et al	2014	The total amount of calculated GHG emissions released into the atmosphere over this seven-year period was 37,000 tonnes of CO₂e, with isoflurane contributing 6%, sevoflurane 17% and desflurane 77% of this total.	24 central public hospitals, 18 regional public hospitals and 23 smaller public hospitals in Victoria, Australia.
(20)	Eggers, B et al	2023	4,172 cases. The nitrous oxide and volatiles produced 48,860 kg of CO₂e. Total anesthesia hours were 5,735 h.	George Regional Hospital, South Africa
(21)	Kim, S et al	2023	2,916 surgical cases were performed in 4000 GA hours. Volatile anesthesia produced 36 tonnes of CO₂.	George Regional Hospital, South Africa.
(22)	Kim, S et al	2022	N₂O > desflurane > isoflurane > sevoflurane are the biggest greenhouse gas contributors. 1,000 consecutive GA hours produced 10,712 kg carbon dioxide equivalent.	George Regional Hospital, South Africa.
(23)	Davies, J et al	2023	38,000 adult surgical procedures annually, in 2019, desflurane constituted 13% of all volatiles purchased, contributed 80% of all volatile anaesthetic GHG emissions. In 2019, desflurane alone contributed 261,664 kg CO₂e.	Austin Health tertiary teaching hospital
(24)	Laverdure, F et al	2013	Between 2006 and 2010, the sevoflurane consumption by act increased by 25%, from 16.5 to 20.6 mL, and desflurane consumption by 37%, from 46.1 to 63.1 mL by patient.	Gustave-Roussy Institute, France.
(25)	Hu, E et al	2024	Across the 5 years (1 January 2017 to 30 June 2022) an average of 242,054 (standard deviation (SD) 16,222) kg of N₂O, equivalent to 64,144 (4,299) tonnes of carbon dioxide emissions (CO₂e), per annum.	Australian hospitals
(26)	Keady, T et al	2023	In 2019, total emissions from inhaled anaesthetic agents used across all public hospitals and the majority of private hospitals amounted to 17,865 tonnes CO₂e or ~0.03% of the total Irish emissions in 2019.	All hospitals (59 hospitals) of the Republic of Ireland.

The diversity of studies on the consumption of halogenated agents in anesthesia extends from the use observed in some countries (26, 27) to the practice of anesthesiologists, where varying emissions arise depending on the anesthetic gas used or the method of airway management, such as with an endotracheal tube, laryngeal mask, or face mask (28, 29).

Other studies focused on *interventions to minimize the environmental impact* of anesthetic gas use. These include the formation of sustainable anesthesia teams, the implementation of educational programs, protocols, and strategies to reduce the use of halogenated gases, the removal of desflurane vaporizers, the use of a new halogenated adsorbent, new anesthesia machines, the promotion of low-flow anesthesia, and the adoption of alternative anesthetic techniques such as total intravenous anesthesia (TIVA) and regional anesthesia (30–46) (Table 2).

**Table 2:** Studies of interventions to minimize the environmental impact of anesthetic agents.

Ref	Authors	Year	Intervention	Results	Centers
(30)	Khan, S et al.	2023	Carbon emissions initiative with Low Fresh Gas Flow Rates.	Reducing the volumes occupied by low flows could decrease emissions of anesthetic gases by 57%.	Creighton University Medical Center
(31)	Bracco, D et al.	2024	Desflurane withdrawal.	Emissions of 23.1 kg CO₂e/h from general anesthesia before desflurane withdrawal, then decreased by 66%, to 7.85 kg CO₂e/h.	Montreal General Hospital
(32)	Glenski, T et al.	2020	Low-flow anesthesia improvement and implementation project.	An estimated reduction of 28.5 MTCO₂e per year was achieved compared to our reference value (114 MTCO₂e).	Adele Hall Campus, Children’s Mercy Hospital of Kansas City
(33)	Hickman, J et al.	2019	EnVol improvement project.	Emissions of 425,280 kg of CO₂e were reduced to 305,670 kg, with an average monthly reduction in CO₂e production of 17,087 kg CO₂e.	University Hospitals Bristol
(34)	Pauchard, JC et al.	2020	Implementation of an anesthesia educational program.	This intervention led to a 51,542 Kg CO₂e decrease for a 3-month period.	University Hospital of Bordeaux
(35)	Minns, J et al.	2023	Educational project to raise awareness of the emissions from general anesthesia.	In North East UK centers, CO₂e emissions decreased by 12.83 tonnes CO₂eq and 7.23 KgCO₂e/h, mainly due to reduced use of desflurane and N₂O.	North East of England and North Cumbria
(36)	Saadat, F et al.	2021	Project Drawdown: reducing the inhalational anaesthetic agents across Wales	The 6 hospitals reduced their CO₂e emissions from 32,008.14 kgCO₂e to an average of 9,892.65 kgCO₂e. The average monthly reduction in CO₂e is 22,115.49 kgCO₂e.	Bangor Hospital, Princess Hospital of Wales, Royal Gwent Hospital, Royal Glamorgan Hospital, University Hospital of Wales and Ysbyty Ystrad Fawr (Large Vale Hospital, Gales).
(37)	Danby, J et al.	2018	Several interventions	The environmental impact of using volatile anesthetics decreased by 15 kg of CO₂e/h.	Freeman Hospital
(38)	Alexander, R et al.	2018	Multiple interventions to reduce emissions.	There was a decrease in the total consumption of volatile anesthetics, especially desflurane. Emissions decreased by 66% (8.9 million kg CO₂e).	Vancouver General/UBC Hospital, St. Paul’s Hospital, Royal Columbian/Eagle Ridge Hospital, and BC Children’s & Women’s Hospital
(39)	Tucker, A et al.	2022	Project Drawdown. Removal of desflurane vaporizers.	During the 5-year study period, a decrease in total CO₂e was observed from 254,280 kg to 46,911 kg, representing a reduction of 81.5%.	Gwyneed Hospital, Wales
(40)	Berwick, C et al.	2022	Volatile awareness- a pan-Mersey quality-improvement project.	Initial emissions of 17,257 KgCO₂e in the 7 days of audit, showed an average reduction from 12.7 KgCO₂e/h to 8.3 KgCO₂e/h.	Countess of Chester Hospital, The Walton Centre and Liverpool University Foundation Trust.
(41)	Chambrin, C et al.	2023	Volatile awareness- a pan-Mersey quality-improvement project.	The average carbon footprint per surgical activity of inhaled halogenated anesthetics was 66.2 kgCO₂e per GA during the first half of 2015 and 6.5 kgCO₂e in the last 6 months.	4 Hospitals of the Hospices Civils de Lyon
(42)	Hudson, S et al.	2022	Quality improvement project	In 2020, there was a reduction in CF due to reduced theatre activity. But desflurane still accounted for 81.6%.	District general hospital, UK.
(43)	Wyssusek, K et al.	2022	Quality improvement interventions such as staff education, distribution of posters and removal of desflurane from OR.	The emissions of desflurane and sevoflurane decreased from 767.16 tonnes of CO₂ e to 92.95 tonnes of CO₂ e, representing a reduction of 87.88%.	Royal Brisbane and Women’s Hospital
(44)	Martindale, T et al.	2016	Education about environmental commitments.	The overall CO₂e has fallen from 4,750 kg to 3,000 kg (37%) and the proportion due to the anaesthetic use of nitrous oxide by 58% over the same time period.	University Hospital Southampton
(45)	Park, E et al.	2024	Educational intervention regarding anesthetic gas emissions	After the education, desflurane use decreased by 50%, whereas sevoflurane use increased by 50%.	Pusan National University Yangsan Hospital and Pusan National University Hospital
(46)	Lawson, C et al.	2019	Different interventions to reduce the use of Desflurane	Total reduction in carbon emissions of 1,499 kg of CO₂e.	Newcastle Upon Tyne Hospitals

*In pediatric patients*, one study assessing intravenous induction found that it significantly reduced emissions in the anesthetic room (47). In adults, two studies have shown that using TIVA as the default technique for general anesthesia could significantly reduce the anesthetic CF (48, 49).

### 3.2 Medical devices or supplies

Recycling waste from the operating room (50) and *reusing medical supplies in hospitals may significantly reduce GHG emissions* (50–73), as shown in Table 3.

**Table 3:** Studies focused on reusing medical devices or supplies.

Reusing may reduce GHG emissions	Ref	Reuse may not consistently result in reduction in GHG emissions	Ref
Surgical caps	(51)	Mechanical prophylaxis of venous thromboembolism	(74)
Gastroscopes	(52)	Vaginal delivery custom packs	(75)
Specula	(53)	Central venous catheter	(76)
Intermittent pneumatic compression	(54)	Anesthetic equipment	(77)
Sharp object containers	(55)	Flexible ureteroscopy	(78)
Laryngoscope handles and blades	(56–58)	Reusable glass jars	(79)
Repair of reusable surgical scissors	(59)	Oxygen consumption	(80)
Laparoscopic surgical instruments	(60)	Instruments for spinal fusion surgeries	(81)
Plastic trays	(61)	Pediatric surgical kit	(82)
Sharp object containers	(62, 63)	Bacterial contamination	(83)
Laryngeal mask airways	(64)	Closed suctioning in ICU	(84)
Pressure cuffs	(65)	ERCP stent	(85)
Trocars for laparoscopic surgery	(66)	Drug administration	(86–89)
Sterilization containers	(67)	Surgical instruments	(90)
Supplies used in neurosurgery	(68)		
Pulse oximeters	(69)		
Ureteroscopes	(70)		
Scrub caps	(71)		
Covers and lift sheets	(72)		
Sleeves	(73)		

Analyzing emissions associated with the reuse of resources, devices, or supplies *can be complex and may not consistently result in reductions in greenhouse gas* [*GHG emissions* due to multiple variables at play (74–90)] (Table 3).

For example, a study by McGain et al. (77) indicated that converting single-use anesthetic equipment to reusable equipment would significantly increase CO_2_ emissions in Australia. In contrast, such a switch in the United Kingdom/Europe could lead to an 84% reduction in emissions, while in the USA, it would result in a 48% reduction. This is due to the origin of each country’s energy matrix.

In addition, there is significant variability in the choice of instruments and supplies among surgeons performing surgical procedures, which could have a significant impact on costs and the environment (90).

### 3.3 Surgical procedures

Several studies have examined *the environmental impact of various surgical procedures* from different perspectives, starting with the waste generated by unused open sutures (91–123), as shown in Table 4.

**Table 4:** Studies focused on examined the environmental impact of various surgical procedures.

Environmental impact of surgical procedures	Ref
Bariatric surgery	(28)
Transcatheter aortic valve replacement procedure	(48)
Unused open sutures	(91, 92)
Cardiac surgery	(93)
Coronary stent	(94)
Vascular surgery	(95)
Endovascular abdominal aortic aneurysm repair	(96)
Adult spinal deformity surgery	(97)
Minimally invasive surgery	(98)
Skin cancer removal	(99, 100)
Carpal tunnel decompression	(101, 102)
Arthroscopic procedures	(103, 104)
Arthroplasty and replacement	(105–109)
Laparoscopic cholecystectomy	(110)
Inguinal herniorrhaphy	(111)
Gastroesophageal reflux surgery	(112)
Abdominoplasty, rhinoplasty and breast augmentation	(113)
Robot-assisted surgeries	(114–118)
Radical prostatectomy	(117)
Transurethral resection of bladder tumors	(119)
Tonsillectomy	(120–122)
Endoscopic sinus surgery	(123)

Some studies have *compared the emissions from medical versus surgical treatments*, such as coronary stent implantation versus coronary artery bypass grafting (94). Other studies have evaluated the environmental cost of medical versus surgical treatment for gastroesophageal reflux disease (112) and concluded that antireflux surgery becomes more effective by the ninth postoperative year.

*In the specific case of ophthalmic surgery*, particularly cataract surgery, it was found that 85% of the CF is attributed to medical equipment supplies (124–128), totaling 67.45 kg CO_2_e per procedure and contributing to an overall hospital emission of 151.9 kg CO_2_e per procedure. In addition, ophthalmology consultations should be considered when calculating total emissions (129–135). A similar pattern is observed in ophthalmology consultations, which also contribute substantially to overall emissions (136).

Again, the *energy matrix of a country* where the procedure is performed and the resources available are also decisive in the CF (102, 137).

### 3.4 Other clinical activities

Various clinical processes and activities within a hospital generate a CF. A variety of areas and procedures have been studied, as described in Table 5.

**Table 5:** Studies that examine the environmental impact of other clinical activities.

Other Clinical Activities:	Ref.
Nephrology	(138, 183–185)
Labor analgesia	(139–143)
Radiology department	(186–195)
Radiotherapy and laser units	(196–198)
Endoscopy units and laryngoscopy	(199–202)
Emergency medical service	(203)
Intensive care units	(144–146)
Management of a hospitalized patient and their diagnosis	(147, 204–206)
Nutritional regimens or diets	(207–209)
Clinical Laboratory	(210–214)

For example:

In *nephrology*, one study comparing the environmental impact of three renal replacement modalities concluded that renal transplantation is the most sustainable option for a 15-year period (138).

In *labor analgesia*, remifentanil PCA produces less CO_2_e than nitrous oxide gas/oxygen and epidural analgesia (139–143). Three studies have examined the emissions in *intensive care units*, revealing that they generate 2.5 times more emissions than basic hospitalization services (144–146).

*Managing patients*, the impact of the COVID-19 pandemic (147) has also been estimated. Although CO_2_ emissions decreased during the pandemic, primarily due to fewer patients in hospitals, CF per patient admission increased significantly.

### 3.5 Support units

Sterilization and laundry are support units for clinical activities within the hospital. The CF was lower when sterilizable instruments were included in sets, with a two- to three-fold increase in emissions when the instruments were managed individually. Additionally, two instrument packaging systems have been compared regarding energy consumption during these procedures (148, 149). Hospital procedures that generate GHG emissions, such as incineration and laundry (150), are also described in greater detail in various studies.

### 3.6 Waste management

Hospitals are significant sources of waste, particularly through emissions from incineration and direct disposal. These impacts can be reduced through active recycling efforts (151–155).

### 3.7 Hospitals

The healthcare system in various countries, including the UK, USA, China, Japan, and Australia, contributes between 2.7 and 8% of the total national emissions. Of these emissions, hospitals are responsible for approximately 39 to 51% (5, 156–158). The differences in CF among these countries are primarily influenced by their energy sources, which include nuclear energy, renewables, or fossil fuels (159–163). However, despite the similarities, each hospital has characteristics that determine the dimensions of the different sources of emissions, energy matrix, organization, processes, and activities (164–167).

The database of results is available in Supplementary material 2.

## 4 Discussion

In this scoping review, 184 studies examining the scope, characteristics, and methodological approaches of the existing literature on the contribution of hospital activities and clinical services to global warming were included. The findings confirm that the healthcare sector is a substantial contributor to national GHG emissions, accounting for approximately 2.7 to 8% of total emissions at the country level. Within this sector, hospitals are responsible for an estimated 39 to 51% of emissions, largely driven by clinical activities and services (5). These results underscore the relevance of hospitals as critical intervention points for climate mitigation within healthcare systems.

The reviewed studies cover a wide range of clinical and support activities. High-impact clinical services such as radiodiagnosis, intensive care, surgery, and anesthesia were frequently examined, alongside support units including sterilization services, laboratory testing, and hospital waste management. Additional topics included recycling practices, dietary interventions, and organizational strategies aimed at reducing hospital carbon footprints. To consolidate this heterogeneous body of evidence, studies were organized according to their primary research focus, facilitating the identification of key areas contributing to hospital emissions: anesthetic techniques, use of medical devices and supplies, operating room procedures, clinical and support activities, and waste generation.

Among these areas, anesthetic practice emerged as one of the most extensively studied contributors to hospital-related emissions. Volatile anesthetic gases are consistently identified as having a high global warming potential and may account for 50% or more of a hospital’s total CF (163). The evidence strongly supports recommendations for the withdrawal of desflurane (31, 39) as established in the Glasgow Declaration by the European Society of Anaesthesiology (2). Furthermore, a growing body of literature has evaluated strategies to reduce anesthetic-related emissions, demonstrating that substantial reductions are achievable without compromising patient care (30–38, 40–42, 44, 45). Table 2. In this context, TIVA is increasingly recognized as a preferred method for adult general anesthesia, offering a significantly lower carbon footprint (49). Importantly, available studies indicate that TIVA does not negatively affect patient outcomes or safety (168), thus reinforcing its role as both a clinically sound and an environmentally sustainable option.

Another prominent theme across the literature is the use of medical devices and consumables, particularly the environmental implications of single-use versus reusable products. The promotion of the “5Rs” of sustainability (reduce, reuse, recycle, rethink, and research) is increasingly evident in healthcare. Many of the included studies used robust environmental assessment methods, most notably the life cycle assessment (LCA) following standards such as ISO 14040 and ISO 14044. Additional frameworks such as the GHG Protocol, ReCiPe, Impact2002+, and sector-specific guides for pharmaceuticals and medical devices were also applied. These methodologies make comprehensive evaluations across the entire life cycle of products possible, from raw material extraction and manufacturing to transportation, use, and disposal or recycling. Such analyses provide evidence-based insights to support decision-making in clinical practice, for example, determining whether the reuse of medical devices effectively reduces GHG emissions compared with single-use alternatives.

Multiple contextual factors influence the environmental performance of reusable versus disposable devices. These include the local energy mix, the number of reuse cycles required to offset initial manufacturing emissions, sterilization efficiency and scale, transportation distances, and end-of-life disposal pathways. For instance, comparative studies of reusable and single-use laryngoscopes demonstrate that conclusions regarding sustainability are highly context-dependent (56). These findings highlight the importance of avoiding generalized assumptions and the need for location-specific assessments.

Despite identifying major emission sources, establishing a universal hierarchy of GHG contributors within hospitals remains challenging. Emission profiles vary substantially depending on local conditions such as supply chains, clinical practices (including anesthetic techniques), and, critically, the energy mix used to power hospital operations. In this regard, the study by MacNeill et al. (163) details the CF of operating rooms in three healthcare systems. The hospitals mentioned, which share similar characteristics, are situated in three different countries. In one, anesthetic gases account for 63% of emissions, while the energy mix (based on renewable energy) only accounts for 17%. At the other extreme, in the third hospital, with low consumption of anesthetic gases and a preference for regional anesthesia and TIVA, this source reached only 4% of total emissions; in comparison, energy consumption represented 84% (based on fossil fuels). Energy consumption is a cross-cutting determinant of emissions across surgical procedures, waste management, resource utilization, and routine clinical activities. Consequently, transitioning hospital energy systems to renewable sources has become one of the most impactful mitigation strategies (5, 156–158, 163). The geographical origin of products and associated transportation requirements significantly influence their CF, as illustrated by studies assessing devices such as laryngeal masks (64).

Several studies in this review also addressed the economic dimensions of hospital-related GHG emissions (23, 50, 56, 57, 59–61, 65, 77, 91, 122, 149). Overall, these analyses suggest that strategies focused on reducing resource use and increasing reuse can lower emissions and costs simultaneously. For example, within operating room processes, the use of cold techniques in adult tonsillectomy has been shown to minimize both economic and environmental impacts (122). This study exemplifies a sustainability assessment that integrates economic, environmental, and social dimensions. Most studies, however, focus on processes or activities without explicitly incorporating standardized measures of clinical effectiveness, such as length of hospital stay, duration of mechanical ventilation, or disability-adjusted life years (DALYs) avoided.

The lack of integrated analyses combining clinical effectiveness, costs, and environmental implications constitutes a major gap in the literature (169). Such integration would support a more comprehensive sustainability assessment, such as estimating emissions or costs per DALY avoided for a given intervention. Incorporating sustainability metrics into clinical trials, clinical practice guidelines, and health economic evaluations is therefore a critical future direction. While methodologically complex and resource-intensive, particularly given the need for multidisciplinary teams and large datasets, such approaches are increasingly necessary given the escalating climate emergency. Clinicians, administrators, and policymakers require tools that enable decisions based not only on effectiveness and cost, but also on environmental impact.

From a life cycle perspective, it is also important to recognize that clinical activities generate environmental impacts that translate into health damage, often expressed in DALYs, through mechanisms such as air pollution, climate change, ozone depletion, and ecosystem degradation. These DALYs generated by healthcare activities must be weighed against the DALYs avoided through the health benefits of interventions (170, 171). For example, while an appendectomy prevents morbidity and mortality, it also generates environmental burdens through resource use. Integrating LCA results with clinical effectiveness data would provide a more balanced assessment of net health benefits.

The review also highlights emerging technologies like robotics and artificial intelligence as areas of both opportunity and concern. Although these technologies have the potential to revolutionize healthcare delivery, their sustainability remains uncertain. High resource consumption, energy demands, and waste generation pose significant challenges (114–118). Ensuring that future technological innovations meet criteria for clinical effectiveness, cost-efficiency, and minimal environmental impact will be essential for their long-term viability.

The broader policy context reinforces the urgency of this agenda. The Declaration of Helsinki 2020 emphasizes the interdependence of human and planetary health, calling on decision-makers to consider environmental consequences alongside health outcomes (6). This call is echoed by the Intergovernmental Panel on Climate Change (172), the Intergovernmental Science-Policy Platform on Biodiversity and Ecosystem Services (173) and the United Nations 2030 Agenda for Sustainable Development (174), which includes several goals directly addressing environmental determinants of health. In this context, the present scoping review responds to an urgent need by synthesizing current knowledge on hospital and clinical contributions to global warming, identifying mitigation strategies, and highlighting priorities for future research.

Compared with previous reviews, this work offers a broader and more up-to-date synthesis of hospital-related emissions. The reviews by Seifer et al. (175) and Alshqaqeeq et al. (176) require updating as they did not encompass the full range of hospital activities. Drew J et al. (177) provided an in-depth analysis of operating room emissions but did not address hospital activities comprehensively. Lenzen et al. (178) conducted a global assessment of the healthcare CF but did not focus specifically on hospital-level or clinical activity contributions.

More recently, Padgett et al. (179) examined methodologies for measuring sustainable healthcare, emphasizing LCA and system-level data, but without identifying specific clinical activities or modifiable emission sources. Similarly, Pickles et al. (180) reviewed clinician- and service-led interventions to reduce healthcare emissions but focused on intervention outcomes rather than mapping emission sources across hospital activities.

This review has several limitations. Notably, the exclusion of grey literature may have resulted in the omission of valuable insights from hospital-based environmental initiatives and professional networks that actively promote sustainable practices (181, 182). Additionally, information available through organizational websites and proprietary databases was not systematically included, potentially limiting the comprehensiveness of the evidence base (171, 172, 174).

## 5 Conclusion

This scoping review provides a comprehensive and structured overview of clinical activities, processes, and resource use within hospitals, organized according to their primary research focus. By synthesizing evidence from multiple sources, it seeks to support clinicians, administrators, policymakers, and researchers in identifying priority areas for action and future investigation, thereby advancing the development of more sustainable healthcare systems amid the climate crisis.
