# Supplementary material for: Contribution of hospitals and clinical services to global warming: a scoping systematic review
Source: Front Public Health. 2026 May 12;14:1778269. doi: 10.3389/fpubh.2026.1778269 (PMC13201132; doi:10.3389/fpubh.2026.1778269)
Supplement: Supplementary file 1 [file Table_1.DOCX]

Supplementary Material 1. Full search strategies for all databases

| **Database** | **Search Term** |
| --- | --- |
| MEDLINE | (((((((carbon footprint) OR (greenhouse effect)) OR (greenhouse gas**)) OR (global warming)) OR (climate change)) OR (life cycle assessment)) AND (((hospitals) OR (hospital units)) OR (laboratories, hospital))) AND ((((((((((((environment** medicine) OR (gases)) OR (anesthesia and analgesia)) OR (Anes- thetics)) OR (Anesthesiology)) OR (Anesthesiologist)) OR (Waste Products)) OR (Sanitary engineering)) OR (Health care)) OR (Health care activities)) OR (Health process)) OR (Medicine)). |
| EMBASE | (((((((carbon footprint) OR (greenhouse effect)) OR (greenhouse gas)) OR (global warming potential)) OR (climate change)) OR (life cycle assess- ment)) AND (((hospital) OR (hospital subdivisions and components)) OR (hospital laboratory))) AND ((((((((((((environmental medicine) OR (gas)) OR (anesthesia procedure)) OR (anesthetics agent)) OR (anesthesiology)) OR (anesthesiologist)) OR (waste)) OR (sanitation)) OR (health care)) OR (health care activities)) OR (health process)) OR (medicine)). |
| SCOPUS | (((((((carbon footprint) OR (greenhouse effect)) OR (greenhouse gases)) OR (global warming)) OR (climate change)) OR (life cycle assessment)) AND (((hospitals) OR (hospital units)) OR (laboratories, hospital))) AND ((((((((((((environment medicine) OR (gases)) OR (anesthesia and analgesia)) OR (Anes- thetics)) OR (Anesthesiology)) OR (Anesthesiologist)) OR (Waste Products)) OR (Sanitary engineering)) OR (Health care)) OR (Health care activities)) OR (Health process)) OR (Medicine)). |
